# Analyzing the Spatiotemporal Distribution and Characteristics of Liver Cirrhosis in Hospitalized Patients in Wuwei, Gansu Province During 1995–2016: A Long-Term Retrospective Study

**DOI:** 10.3389/fphys.2022.845095

**Published:** 2022-03-22

**Authors:** Yang Zhang, Ting Fu, Xiao-Jie Yuan, Yan-Cheng Ye, Zhi-Wen Guo, Kun Liu, Zhao-Hua Ji, Zhong-Jun Shao

**Affiliations:** ^1^ Department of Epidemiology, School of Public Health, Air Force Medical University, Xi’an, China; ^2^ Shaanxi Energy Institute, College of Nursing, Xi’an, China; ^3^ Ministry of Education Key Lab of Hazard Assessment and Control in Special Operational Environment, Xi’an, China; ^4^ Gansu Wuwei Tumour Hospital, Wuwei, China; ^5^ Department of Infection Disease Control and Prevention, Wu Wei Center for Disease Prevention and Control, Wuwei, China

**Keywords:** liver cirrhosis, temporal trend, spatial trend, spatial-temporal, cluster

## Abstract

**Objectives:** This was a long-term retrospective study, aiming to understand the temporal and spatial trend of cirrhosis in Wuwei from 1995 to 2016, explore its spatio-temporal aggregation, and find out the high incidence areas. To provide theoretical basis for the formulation of comprehensive prevention and treatment strategy of cirrhosis in Wuwei.

**Methods:** Herein, we extracted data of cirrhosis patients who were treated in 12 sentinel hospitals in Wuwei from their medical records. We used SAS and Joinpoint Regression Program for data analysis, SaTScan 9.4 software for clustering area detection, and ArcGIS 10.2 software for geographical distribution mapping.

**Results:** Among 3308 patients with liver cirrhosis (average age, 55.34 years) included in this study, 15.9% were aged 50–54 years. The majority were men (2716, 65.8%), with a sex ratio of 1.92:1 and peasants by occupation (1369, 60.3%). The basic social medical insurance system covered the healthcare costs of 1271 patients (63%). A Joinpoint regression analysis done for 1995–2016 revealed an increase in the standardized cirrhosis rate [average annual percent change (AAPC) = 16.7% (95% CI, 10.2–23.5%)] with three joinpoints in 2010, 2013, and 2016. The annual percent change (APC) from 1995 to 2010 was 11.13% (95% CI: 6.5–16.0), and APC from 2010 to 2013 was 66.48% (95% CI:16.0–138.9); conversely, from 2013 to 2016, APC was 4.4% (95% CI, −7.5–17.8%). Hongshagang Town showed the highest average incidence. Each township showed a gradual increase in the incidence after 2010. The results revealed that in each township, liver cirrhosis incidence had some spatial aggregation and was nonrandom. Four liver cirrhosis clusters were noted in 75 townships in Wuwei. Data were gathered from 2011 to 2016.

**Conclusions:** From 1995 to 2016, the incidence of cirrhosis in Wuwei still showed an increasing trend, but the growth rate slowed down since 2013. In Wuwei, the rate of standardization of cirrhosis in female patients increased steadily and faster than in male patients. It is necessary to strengthen the diagnosis, treatment, prevention, and control measures of cirrhosis-related diseases. The results of spatial scanning, basic spatial distribution, aggregation time, and time trend analysis were consistent.

## Introduction

In clinical practice, liver cirrhosis is a commonly encountered chronic disease. It is commonly caused by viral hepatitis, chronic alcoholic liver damage, cholestasis, nonalcoholic steatohepatitis, industrial poisons or drugs, and autoimmune hepatitis. However, the pathogenesis of cirrhosis shows obvious regional differences. Viral hepatitis B has high prevalence in China, and chronic hepatitis B is the main cause of liver cirrhosis. Conversely, in developed Western countries of Europe and America, alcoholic cirrhosis is the main cause of liver cirrhosis (de Franchis and Primignani, 2001). Hepatitis, cirrhosis, and liver cancer are a triad of closely related liver diseases. According to the World Gastroenterology Organisation global guidelines, 1%–8% of decompensated liver cirrhosis, 6% of active compensatory liver cirrhosis, and 2% of nonactive liver cirrhosis will turn into liver cancer each year. Globally, between 500,000 and 700,000 people die from cirrhosis every year, accounting for 2.7% of all deaths. This makes it the fifth most predominant cause of death in developed countries (Kondili et al., 2005; Zatoński et al., 2010). According to the WHO (World Health Organization, WHO), cirrhosis affects an average of 17.1 per 100,000 people worldwide, and its incidence is on the rise. Notably, the incidence of cirrhosis is higher in Africa, Asia, and other areas with high incidence of viral hepatitis. (Lavanchy, 2004). Therefore, understanding the epidemiological characteristics of liver cirrhosis is important (e.g., geographical factors, population distribution, and temporal changes).

Herein, we analyzed the data of patients treated for liver cirrhosis in 12 sentinel hospitals of Wuwei City between 1995 and 2016 with an aim to study the disease and demographic characteristics and the temporal epidemiological and spatial trends in these patients. We compared the differences in temporal and spatial trends and disease characteristics among patients having liver cirrhosis in Minqin County, Liangzhou District, Tianzhu County, and Gulang County. We also explored the factors causing these differences.

## Methods

### Data Sources

In this retrospective study, the morbidity data included were extracted from inpatient medical records of 12 sentinel hospitals (secondary level or above) of Wuwei City, Gansu Province from 1995 to 2016. If a single patient had repeated diagnoses at multiple hospitals, either simultaneous or sequential, we considered only one medical record in the analysis. The joinpoint time trend analysis used population data from the 2010 sixth national census and the 2016 census data released by the statistics bureau of Wuwei City.

### Data Quality Control Methods

In the research process, the paper medical records were photographed by the staff to generate PDF files for archiving, and the electronic medical records were directly exported to form electronic documents. Supervisors and randomly selected team members arbitrarily checked 10% of PDF documents at all levels of hospitals every day to check for omissions in medical records. The supervisor checked daily whether the collected medical records were consistent with the number of recorded cases.

### Statistical Methods

Joinpoint Regression Program (Statistical Methodology and Applications Branch, Surveillance Research Program, National Cancer Institute) and SAS (SAS Institute, Inc., Cary, NC, United States) were used for data analyses. For geographical distribution mapping, we used the ArcGIS 10.2 software (ESRI, Redlands, CA, United States), and for clustering area detection, we used SaTScan 9.4 software (Kulldorff M. and Information Management Services, Inc.). The SAS software was also used to analyze general data pertaining to hospitalizations, demographic characteristics, and treatment. Categorical variables were presented as numbers, composition ratios, and frequencies; continuous variables were described as mean ± standard deviation (SD). We used the Fisher’s exact test or *χ* ^*2*^ test for between-group comparisons, and *p* < 0.05 represented a statistically significant difference.

The crude incidence rate was standardized by the standard population composition of the sixth national census in 2010. During 1995–2016, the joinpoint software was used to analyze the standardized rate, percentage rate, time trends, and age group rates of liver cirrhosis in rural regions of Wuwei City. We calculated the annual percentage change (APC) as well as its 95% confidence interval (CI) values. We used the joinpoint regression model and the grid search method for fitting the models and judging statistically significant trends, respectively, and we used multiple permutation tests for identifying join points with significant differences in 95% CI values and APC for each trend (Doll and Cook, 1967; Kim et al., 2000; Lee et al., 2018). The two major advantages of the joinpoint software are as follows. First, the increase or decrease in disease incidence was estimated by calculating APC and 95%CI for each trend phase. Second, it identifies the year of change in direction of the trend (Reas and Rø, 2018). To enable direct comparison between different age groups, the weighted average APC (AAPC) could determine the overall time trend. APC was considered equal to AAPC in the absence of a change in trend direction (i.e., no join point). For describing the trends with a statistically significant trend slope (APC) (*p* < 0.05), the words “increase” or “decrease” were used. Conversely, for describing statistically insignificant trends, the word “stable” was used (Bray et al., 2018).

We considered the lower-left corner of the Wuwei City map as the origin, and for the distribution map, we prepared a two-dimensional space Cartesian coordinate system. Then, to construct the geographic information system, the liver cirrhosis incidence database of the 12 sentinel hospitals in Wuwei City for the period 1995–2016 was associated with the map. Depending on the average annual incidence rate of liver cirrhosis, we used different colors for each region. For the aforementioned period, the spatial distribution map of liver cirrhosis in 102 towns and villages in Wuwei City could be drawn continuously to describe the spatial annual distribution characteristics of liver cirrhosis and the change in trend over 22 years. Then, we used the SaTScan 9.4 software to perform spatial clustering analysis for spatiotemporal scanning. We used a series of scanning circles to detect the existence and location of clustering in the study area. We determined any statistically significant difference between the two groups as well as the relative risk (RR) of the aggregation area (Jonsson et al., 2010).

### Ethical Approval

Each procedure performed in this study followed the ethical standards of the Air Force Medical University Ethics Committee and was in compliance with the tenets of the 1964 Helsinki declaration and its later amendments or comparable ethical standards.

## Results

### Baseline Characteristics of Inpatients With Liver Cirrhosis

The mean age (SD) of the 3308 patients was 55.34 ears (12.552 years), with the age group of 50–54 years affected the most, followed by age groups of 60–64 years, 45–49 years, and 55–59 years. Most patients in Liangzhou District and Minqin County were aged 50–54 years. Conversely, most patients in Gulang County were aged 60–64 years. Tianzhu County showed the same distribution of cases of patients with age groups of 45–49 years and 50–54 years.

Overall, there were a total of 2176 men (65.8%), and their distribution was as follows: Liangzhou District, 1492 (66.1%); Minqin County, 233 (63.8%); Gulang County, 326 (65.2%), and Tianzhu County, 125 (67.6%). More men than women were hospitalized with liver cirrhosis in the 12 sentinel monitoring hospitals in Wuwei; the highest and lowest male:female patient gender ratios noted were 11:1 and 1.55:1. Trend changes in the proportion of hospitalized men and women were statistically significant (χ^2^ = 22.712, *p* < 0.001). Table 1 shows changes in sex ratio and distribution from 1995 to 2016.

**TABLE 1:** The sex distribution and ratio of inpatients with liver cirrhosis during 1995–2016.

Year	Male	Percent(%)	Female	Percent(%)	Total	Sex ratio
1995	11	0.51	1	0.09	12	11.00
1996	13	0.60	3	0.27	16	4.33
1997	21	0.97	7	0.62	28	3.00
1998	17	0.78	5	0.44	22	3.40
1999	11	0.51	5	0.44	16	2.20
2000	23	1.06	8	0.71	31	2.88
2001	33	1.52	16	1.41	49	2.06
2002	37	1.70	13	1.15	50	2.85
2003	16	0.74	10	0.88	26	1.60
2004	41	1.88	15	1.33	56	2.73
2005	55	2.53	20	1.77	75	2.75
2006	55	2.53	20	1.77	75	2.75
2007	49	2.25	11	0.97	60	4.45
2008	59	2.71	23	2.03	82	2.57
2009	35	1.61	16	1.41	51	2.19
2010	39	1.79	31	2.74	70	1.26
2011	152	6.99	57	5.04	209	2.67
2012	208	9.56	119	10.51	327	1.75
2013	266	12.22	141	12.46	407	1.89
2014	353	16.22	191	16.87	544	1.85
2015	334	15.35	195	17.23	529	1.71
2016	348	15.99	225	19.88	573	1.55
**Total**	2176	100.00	1132	100.00	3308	1.92

Among the patients hospitalized for cirrhosis, the ethnicities of 3258 (98.5%), 39 (1.2%), 7 (0.1%), and 4 (0.1%) patients were Han, Tibetan, Tu, and Hui, respectively. Specifically, these statistics for Liangzhou District were 2219 (98.2%), 30 (1.3%), 3 (0.1%), and 6 (0.3%), respectively, and for Gulang County were 492 (98.4%), 6 (1.2%), 1 (0.2%), and 1 (0.2%), respectively. Minqin County had 2 (0.5%) Tibetan and 363 (99.5%) Han patients, while Tianzhu County had 1 (0.5%) Tibetan and 184 (99.5%) Han patients. Overall, 1795 patients (54.3%) were peasants, 492 (14.9%) were workers, 388 (11.7%) were cadres, 8 (0.2%) were students, 42 (1.3%) were freelancers, 187 (5.7%) were unemployed, and 396 pursued other occupations (11.9%). On statistical analysis, the incidence of liver cirrhosis was found to significantly differ among patients with different occupations (*p* < 0.05). During 1995–2016, the occupational proportion of hospitalized patients with liver cirrhosis in Wuwei City was still the highest for peasants; however, in recent years, the proportion of workers, freelancers, and individuals with other occupations has increased (*χ* ^*2*^ = 11.931, *p* < 0.001).

**FIGURE 1:**
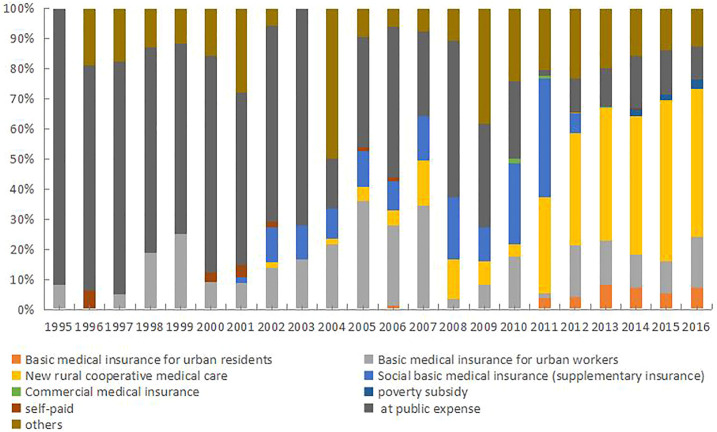
Healthcare financing methods used by patients during 1995–2016.

A total of 2389 (72%) patients availed their basic social medical insurance for payments. This insurance system includes but is not limited to the basic medical insurance for urban residents and employees, the new rural cooperative medical care system (NRCMS), and basic social medical insurance (supplementary insurance). Overall, 348 patients (11%) paid out of their pockets, 41 (1%) had poverty subsidy, 9 (0.3%) were treated at public expense, and 5 (0.15%) had private insurance, respectively. Figure 1 shows the changing trends in healthcare financing methods used for settling hospitalization expenses by patients with liver cirrhosis in Wuwei City over the past 22 years; this also reflects a change in China’s social security system.

### Analysis of the Temporal Trends in Incidence of Liver Cirrhosis

Table 2 shows that according to joinpoint regression analysis, the overall standardized liver cirrhosis rate increased from 1995 to 2016, with an average rate of increase of 16.70% per year (95% CI, 10.2–23.5%) and join points in 2010 and 2013. Although the standard incidence of liver cirrhosis rapidly increased after 2010, the increase was less rapid after 2013. The change in APC among men and women was statistically significant from 1995 to 2010 (APC = 11.1% [95% CI, 6.5–16.0%]) and from 2010 to 2013 (APC = 66.5% [95% CI, 16.0–138.8%]). From 2013 to 2016, APC was 4.4% (95% CI, −7.5–17.8%).

**TABLE 2:** The APC/AAPC and age-standardized rate of liver cirrhosis from 1995 to 2016.

Year of diagnosis	Male and Female		Male		Female	
	Age-Adjusted rate	APC 95%CI	Age-Adjusted rate	APC 95%CI	Age-Adjusted rate	APC 95%CI
1995	0.82	—	1.48	—	0.13	—
1996	1.15	—	1.83	—	0.43	—
1997	1.82	—	2.45	—	1.12	—
1998	1.76	—	2.74	—	0.80	—
1999	1.18	—	1.50	—	0.86	—
2000	1.91	—	2.83	—	0.99	—
2001	3.04	—	4.21	—	1.85	—
2002	3.27	—	4.60	—	1.93^a^	25.7^b^ (5.2–50.1%)
2003	1.76	—	2.08	—	1.54	—
2004	3.54	—	5.07	—	1.95	—
2005	4.86	—	7.03	—	2.52	—
2006	5.03	—	7.87	—	2.42	—
2007	3.58	—	5.87	—	1.38	—
2008	4.93	—	7.14	—	2.71	—
2009	3.03	—	4.32	—	1.92^a^	0.3 (−12–14.5%)
2010	4.19^a^	11.1^b^ (6.5–16%)	4.83^a^	10.9^b^ (6.3–15.8%)	3.60	—
2011	12.97	—	18.68	—	7.33	—
2012	19.28	—	24.06	—	14.62^a^	92.2^b^ (20.5–206.6%)
2013	24.97^a^	66.5^b^ (16–138.9%)	32.34^a^	60.7^b^ (8.9–137%)	16.85	—
2014	31.07	—	40.06	—	21.92	—
2015	31.08	—	37.90	—	23.26	—
2016	32.25	4.4 (−7.5–17.8%)	38.94	2.4 (−10.4–17%)	26.44	16.3^b^ (8.3–24.9%)
AAPC 95%CI	16.7^b^ (10.2–23.5%)	—	15.6^b^ (8.8–22.8%)	—	22.1^b^ (11.6–33.5%)	—

a Indicates there is 1 joinpoint point in the year.

b Indicates that at the alpha = 0.05 level, APC/AAPC, is significantly different from zero.

AAPC, average annual percent change; APC, annual percentage change; CI, confidence interval.

The average standardized rate of increase of liver cirrhosis in men was 15.6% per year (95% CI, 8.8–28.8%). There was a join point in 2010 (a significant turning point indicative of rapid growth). The change in APC among men was statistically significant from 1995 to 2010 (APC = 10.9% [95% CI, 6.3–15.8%). There was also a join point in 2013 (a significant turning point indicative of slowdown). The change in APC among men was statistically significant from 1995 to 2010 [APC = 60.7% (95% CI, 8.9–137%)], and APC from 2013 to 2016 was 2.4% (95% CI, –24.4–17%].

**FIGURE 2:**
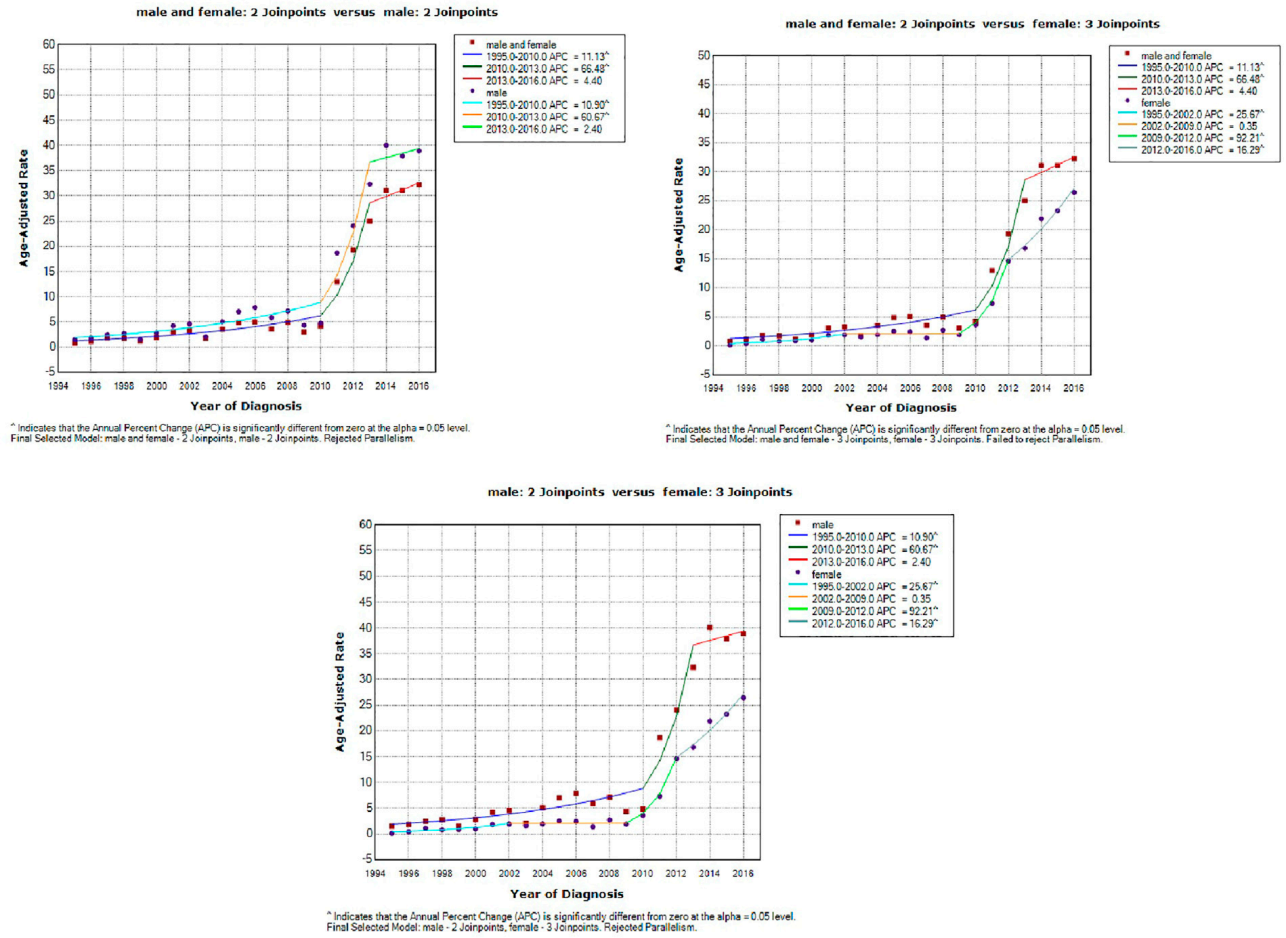
The time trend in liver cirrhosis incidence in all groups and both sexes from 1995 to 2016.

The average standardized rate of increase of liver cirrhosis in women was 22.1% per year (95% CI, 33.5–4.4%), with three join points in 2002, 2009, and 2012. The change in APC among women was statistically significant from 1995 to 2002 [APC = 25.7% (95% CI, 5.3–50.1%)]. The change in APC among women was statistically significant from 2002 to 2009 (APC = 0.3% [95% CI, −12–14.5%]). The change in APC among women was statistically significant from 2009 to 2012 [APC = 92.2% (95% CI, 20.5–206.6%). The change in APC among women was statistically significant from 2012 to 2016 [APC = 16.3% (95% CI 8.3%–24.9%)]. Figure 2 shows the time trend in liver cirrhosis incidence from 1995 to 2016.

### Spatial Trend and Spatiotemporal Clustering Analysis of Liver Cirrhosis at the Township Level

**FIGURE 3:**
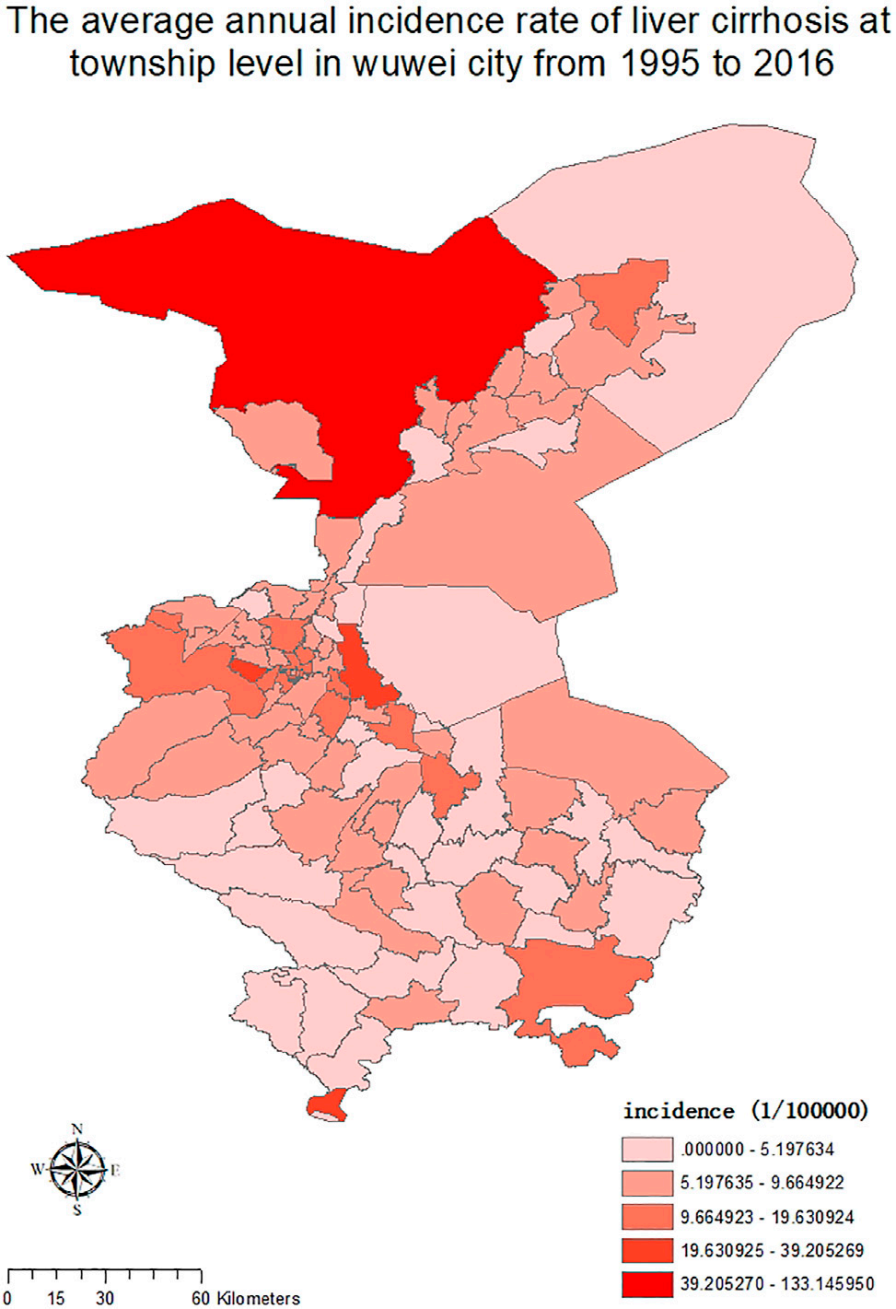
The township-level distribution of the average annual incidence of liver Cirrhosis in Wuwei City during 1995–2016.

**FIGURE 4:**
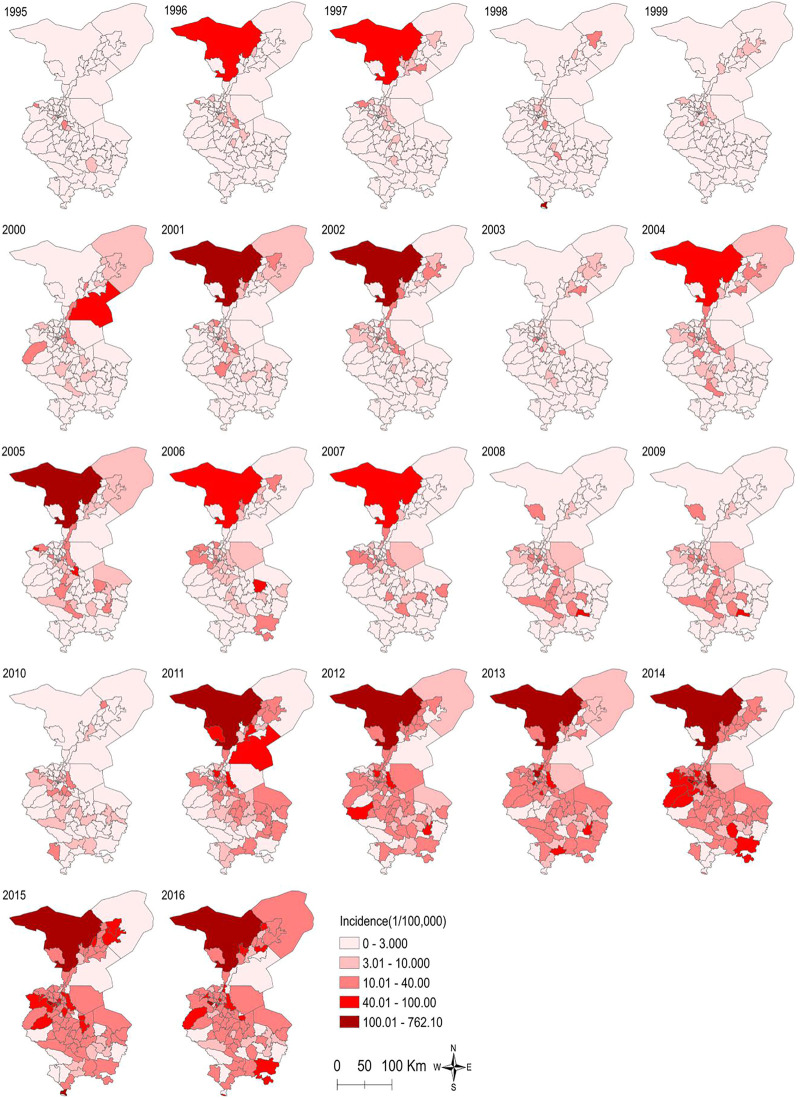
The incidence of liver Cirrhosis at the township level in Wuwei City from 1995 to 2016.

In 102 townships in Wuwei City, the average annual standardized incidence rate of liver cirrhosis was divided into 5 grades using the ArcGIS software, and as shown in Figure 3, the increasing trend from low to high incidence was represented by different color shades. Consequently, the incidence distribution in townships revealed that Hongshagang Town, Minqin County (133.146/100,000) had the highest annual incidence. The annual incidence rates of Songshu Township in Liangzhou District, Celalong Township in Tianzhu County, Qingyuan Town in Liangzhou District, Yongchang Town, Jinshan Township, and Baishu Township were 39.20527/100,000, 37.1563/100,000, 26.04738/100,000, 19.63092/100,000, 16.52544/100,000, and 15.5607/100,000, respectively. There were no cases in Huahuatan Township, Gulang County. Notably, the average annual incidence in Xinbu Township, Hengliang Township, and Tshanshanling Town was relatively low at 1.724739, 1.648257, and 1.425851/100,000, respectively. Figure 3 and Figure 4 show the incidence of liver cirrhosis in Wuwei City during 1995–2016.

**FIGURE 5:**
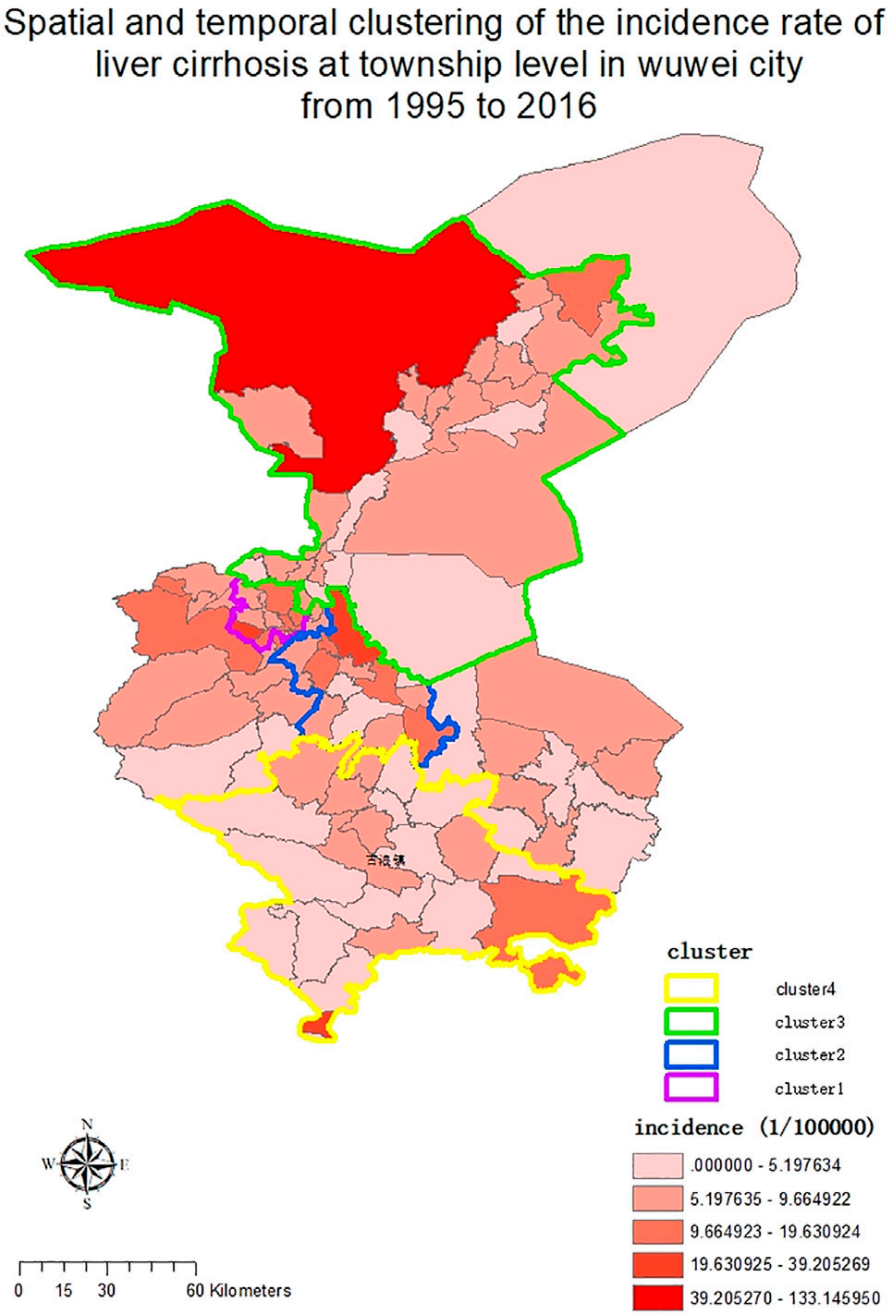
Township-level spatial and temporal clustering of liver cirrhosis incidence in Wuwei City during 1995–2016.

We used SaTScan and ArcGIS 10.2 softwares for the detection of the spatiotemporal clustering of liver cirrhosis incidence in Wuwei City. Four clusters were identified in the study period. Having a maximum scanning radius of 9.70 km, the first cluster covered 14 towns. This cluster showed predominant distribution in Liangzhou subdistrict offices (e.g., railway station, Zhongba, Yongchang, Jinyang, and Xiguan). For the clustering of time during 2012–2016, the log-likelihood ratio (LLR) was 671.692142 and RR was 6.10 (*p* < 0.01). Having a maximum scanning radius of 24.65 km, the second-class aggregation area covered 14 towns. This showed predominant distribution in Liangzhou subdistrict offices (e.g., Huangyanghe, Wujiajing, Donghe, and Hedong). The clustering of time was from 2012 to 2016, and the LLR was 279.561620 and RR was 3.76 (*p* < 0.01). The third cluster covered twenty-five towns (e.g., Sanlei Town, Suwu Town, and Daba Town of Minqin District). The maximum scanning radius was 75.88 km; for the agglomeration time of 2011–2016, the LLR was 85.459553 and RR was 2.23 (*p* < 0.01). Finally, with a maximum scanning radius of 62.13 km, the fourth cluster covered 22 towns with the majority of distribution in the towns of Tianzhu County (e.g., Tanshanling, Tiantang, and Saisishi). For the clustering of time from 2013 to 2016, the LLR was 44.569697 and RR was 2.05 (*p* < 0.01). Table 3 and Figure 5 show the details in this regard.

**TABLE 3:** Spatiotemporal hotspots of liver cirrhosis in Wuwei City, Gansu Province, China, 1995–2016.

Hotspots	1	2	3	4
Time period	2012/1/1 to 2016/12/31	2012/1/1 to 2016/12/31	2011/1/1 to 2016/12/31	2013/1/1 to 2016/12/31
Number of observed cases	768	515	368	227
Number of expected cases	157.64	155.43	176.19	114.86
RR	6.10	3.76	2.23	2.05
LLR	671.692142	279.561620	85.459553	44.569697
Annual incidence	40.8	27.7	17.5	16.5
Number of counties within hotspot	14	14	25	22
Population within the hotspot	340802	334346	346190	326855
Central point/radius	(37.988600 N, 102.576000 E)/9.70 km	(37.774900 N, 102.920000 E)/24.65 km	(38.632300 N, 103.089000 E)/75.88 km	(36.984300 N, 102.717000 E)/62.13 km

RR = Relative risk for the liver cirrhosis incidence in the hotspot compared to the average incidence at the same time period; LLR = Log likelihood ratio.

## Discussion

### Baseline Characteristics of Inpatients With Liver Cirrhosis

Herein, 3308 patients qualified on the basis of enrolment requirements. Their average age (SD) was 55.34 years (12.552 years). Overall, the age group of 35–78 years comprised the majority of cases, within which the age group of 50–54 years had the largest number of cases, which was consistent with the research results of many domestic scholars (Castro et al., 2014). The study results showed that the risk of liver cirrhosis is higher in the older age groups. With China’s economic and social development, improvements in living standards and medical technology, and the consequent increase in the life span, the risk of liver cirrhosis has trended toward older age groups. With a sex ratio of 1.92:1 (which fluctuated during the study period, the incidence of liver cirrhosis was lower in women than in men. According to the results of the trend chi-square test, during our study period, there was a statistically significant difference in the trend change of the proportions of men and women, with the proportion of women showing an increasing trend during this period. From 1995 to 2016, the highest men:women sex ratio was 11:1; overall, it predominantly fluctuated between 4.45:1 and 1.55:1, which is lower than the ratio previously reported in the results of a similar study done for the British population, which ranged from 3.6:1 to 8:1 (Leon and McCambridge, 2006).

According to the results of several Chinese and international researches, the causes of liver cirrhosis are viral hepatitis, alcoholic liver cirrhosis, nonalcoholic liver diseases, obstruction of the hepatic vein, and genetic, metabolic, or autoimmune liver diseases. Viral hepatitis B is endemic in China; it is also the most prominent cause of liver cirrhosis (Schütz et al., 2018). The main reason for it being more prevalent in men than in women is the dual influence of hepatitis B and alcoholic liver disease in men; however, the proportion of women having liver cirrhosis is increasing. The main causes of liver cirrhosis in women are autoimmune liver disease and hepatitis B; in particular, nonalcoholic fatty liver disease (NAFLD) caused by metabolic factors has attracted a lot of attention. Some foreign studies (Rodrigues et al., 2014) show that the prevalence of NAFLD is higher in women, particularly during menopause, which may be associated with reduced estrogen levels after menopause. A decade-long study by Japanese scholars showed an increase in the prevalence of NAFLD in postmenopausal women from 6% before menopause to 15% after menopause (Hamaguchi et al., 2012). Therefore, it is necessary to focus on other causes of liver cirrhosis besides viral hepatitis to reduce the occurrence of liver cirrhosis and implement stronger prevention and control measures.

Wuwei City is located in the middle of Gansu Province and is the east gate for the silk road entering the Hexi Corridor and Xinjiang from east to west. There are 38 ethnic groups in this region, including Han, Tibetan, Hui, Mongolian, and Tu. The Han ethnicity accounts for 98.5% of the population, followed by Tibetans at 1.2%, Tu at 0.2%, and Hui at 0.1%. The results of a large-scale study on the prevalence of hepatitis B in the population and its influencing factors in the Wuwei region showed that at 7.40%, the HBsAg positivity rate of the Han population was statistically significantly higher than that of the minority populations. The rural population (peasants) accounted for 54.3% of the population, followed by workers at 14.9%, others at 11.9%, and cadres at 11.7%. From 1995 to 2016, the proportion of workers, cadres, and individuals in other occupations has increased. According to the chi-square test results of the trend, the proportion of workers with liver cirrhosis has increased over time. This may be explained by urbanization and industrialization, which may have caused many workers to leave the countryside to work in various industries.

A total of 2389 patients (72%) paid their hospital bills through the basic social medical insurance system, and the payment mode of hospitalized patients with cirrhosis has seen changes from 1995 to 2016 (Figure 1). Before 1998, the mode of payment was either out of pocket, public expense, or other ways. In 1998, China began to implement the urban workers’ medical insurance system. Because Wuwei City is dominated by the agricultural population, the rate of use of the urban workers’ medical payment method is not high. In 2003, the NRCMS was officially implemented. After 2009, the proportion of patients under the NRCMS insurance increased significantly. With the ongoing improvements in China’s social security system, the use of the social medical insurance system greatly alleviates patients’ economic burden.

### Temporal Trend

The overall standardized liver Cirrhosis rate has seen an increase at an average rate of 16.7% per year (95% CI, 10.2–23.5%) from 1995 to 2016 according to the joinpoint regression analysis, with two join points in 2010 and 2013. The population growth rate from 2010 to 2013 was about six times higher than that from 1995 to 2010. The change in this trend may be related to the change in the etiology and composition of liver cirrhosis. The etiology of liver cirrhosis has been quite different in China from that in other countries. According to a research done in France, 66.6, 16.0, 14.7, and 2.7% of liver cirrhosis cases were due to alcohol, hepatitis C virus infection, alcohol concurrent with chronic hepatitis virus infection, and hepatitis B virus infection, respectively (Moreau et al., 2004). Liver cirrhosis is common in China due to hepatitis C cirrhosis, hepatitis B cirrhosis, autoimmune liver cirrhosis, and alcoholic cirrhosis. According to the research results of Chinese scholars, the incidence of hepatitis B in Wuwei showed an increasing trend from 1990 to 2016 (*Z*=132.75,*p* < 0.01), 21.32/100 000 in 1990, the highest in 2008 was 746.73/100 000, the average annual incidence was 281.00/100 000, increasing by 21.84%; After 2008, the incidence decreased year by year to 46.82/100,000 in 2016.The time nodes of change are basically the same as that of this study (Ye and Guo, 2018). The number of patients with cirrhosis and other chronic liver diseases caused by hepatitis B increased by 79.6% and the prevalence increased by 49.2% in China in 2016 compared with 1990, according to another scholar (Zhang et al., 2020). However, with the changes in lifestyle and strengthening of the prevention as well as control measures of infectious diseases, the etiological composition of cirrhosis in China is now relatively more similar to that in other countries (Bao et al., 2015). The free vaccination program for hepatitis B has significantly reduced the rate of children infected with hepatitis B virus in China. In addition, the effective use of antiviral drugs and interferon in treating hepatitis B has reduced the incidence of hepatitis B cirrhosis (Liang et al., 2009). This partly explains the reduction in the increase rate of cirrhosis after 2013. The increased prevalence of alcoholic liver disease in China from 2010 to 2013 may be related to the increased population growth rate in that period. The proportion of patients hospitalized for alcoholic cirrhosis increased gradually from 10.8% in 1999 to 24% in 2004. This period also saw the development of medicine and improvements in diagnostic and treatment techniques, resulting in early diagnosis and higher detection rate of liver diseases, which may be related to the significantly accelerated rate of liver cirrhosis standardization during this period.

Liver biopsy has always been regarded as the “gold standard” in the diagnosis of liver fibrosis. However, it has many defects, and its application in clinical work is greatly limited, and patients’ compliance is poor, which to some extent causes difficulties and errors in disease diagnosis (Lu and Hu, 2013). Since the early 1980s, the determination of serum markers of liver fibrosis has been applied in clinical practice, bringing great progress to the diagnosis of liver cirrhosis. In the 1990s, liver cirrhosis was diagnosed by serum markers of liver fibrosis (such as HA,LN, procollagen type III, type IV collagen), color doppler ultrasonography and pulse Doppler ultrasonography (Zheng et al., 2002; Zhang and Zhu, 2005). In 2010, with the progress of medical imaging technology, especially the development of ultrasound elastic detection technology and magnetic resonance technology, their sensitivity and specificity for the diagnosis and grading evaluation of liver fibrosis have been improved. At the same time, the single test for early cirrhosis is difficult to reflect the presence of cirrhosis, even until the decompensated stage of cirrhosis. In recent years, the combined application of different clinical tests and evaluation indexes has established a non-invasive comprehensive index diagnostic model for liver fibrosis, which has gradually become a research hotspot in the diagnosis of liver fibrosis (Wai et al., 2003; Kim et al., 2007).

During our study period, the standardized liver cirrhosis rate in male patients increased at an average rate of 15.6% annually (95% CI, 8.8–28.8%). The incidence rate significantly differed between men and women and was higher in men than in women, which is closely related to the etiology of cirrhosis. The incidence of hepatitis B and alcoholic liver disease was higher in men than in women. In addition, smoking, drinking, irregular diet, and other harmful habits were more prominently present in men, leading to a higher incidence of liver cirrhosis in men. Based on the findings of time trend analyses, the time trend of the standardization rate of cirrhosis in men is consistent with the general trend. During our study period, the changing trend of the standardization rate of liver cirrhosis in women showed a large fluctuation and increased at an average annual rate of 22.1%, with three join points in 2002, 2009, and 2012. In the two periods with statistically significant APC trends, the rate of liver cirrhosis increased faster in women than in men. This indicates that although the incidence is higher in men than in women, the incidence in women has a significant increasing trend, and therefore, it is crucial to not ignore the prevention and treatment of liver cirrhosis in women. This increase may be related to the increased prevalence of autoimmune liver diseases (e.g., NAFLD and primary biliary liver disease) (Acalovschi, 2014). The advancements in medical technology and liver biopsy techniques complemented by an increased understanding of autoimmune liver disease, NAFLD, and other diseases as well as better healthcare awareness may lead to higher early detection rates of non-viral liver disease and reflect as increased prevalence rate.

### Spatial Trend and Spatiotemporal Clustering Analysis

Geographically, regions with a high and low incidence of liver cirrhosis are typically adjacent to each other. This indicates that since there still are high- and low-incidence areas for the occurrence of liver cirrhosis, there may be some potential risk factors in the high-incidence areas contributing to the disease. As shown in Figure 5, before 2010, liver cirrhosis had overall low incidence rates, with few townships, such as Hongshagang Town in Minqin County, reporting a continuously high incidence rate of liver cirrhosis. From 2011 to 2016, the darker areas on the map expanded. This trend is consistent with the results of joinpoint’s time trend analysis. Hongshagang Town of Minqin County has the highest annual incidence rate. This area is located in the northwest direction of Wuwei city. According to local demographic data, the total population of Hongshagang Town in Minqin County has been on a declining trend since 1995 due to ecological migration and relocation. In 22 years, the total population of Hongshagang town decreased from 1,583 in 1995 to 655 in 2016. Due to the backward local economic conditions and harsh natural environment, most of the population moved to the county and urban areas. However, while the population base has decreased, the data show that the number of cirrhosis cases in the region has continued to increase. The decrease of population base and the increase of new cases make the incidence of cirrhosis in this region significantly higher than other regions.

Based on the spatiotemporal detection results, there is an evident aggregation of liver cirrhosis cases in Wuwei City. There are four aggregation areas, and the aggregation time distribution is from 2011 to 2016, which is also mutually confirmed by the time trend analysis results. Combined with the etiology of cirrhosis, according to the analysis of the spatial epidemiological characteristics of viral hepatitis B from 2005 to 2014 by other scholars in China, the incidence rate of hepatitis B declined from 2005 to 2014, which also reflected the nationwide efforts to prevent and control hepatitis B. At the same time, hepatitis B incidence appeared as clustered in some prefecture-level cities (Conte et al., 2011). Gansu Province was a high-incidence area from 2005 to 2009 and from 2010 to 2014, thus suggesting a regional vulnerability to high incidence of hepatitis B. Therefore, prevention and control policies should not be controlled by administrative divisions but should rather be formulated into regional prevention and control policies. The incidence of hepatitis C, another common cause of cirrhosis, also appeared clustered at prefectural and municipal levels. The transmission route and prevention and control measures of hepatitis B and C are similar. However, other causes of cirrhosis, such as alcoholic liver disease, autoimmune liver disease, and NAFLD, have obvious ethnic, regional, and gender differences. In recent years, the etiology of cirrhosis in China has changed with the growing Western lifestyle, which has caused increased prevalence of metabolic disorders, such as obesity, non-viral liver disease, and diabetes (Reynolds et al., 2007).

## Limitations

This study was a long-term retrospective study. Since many available research materials had restricted access, we could not obtain some information, particularly from the early stages of medical records data collection; therefore, some data may be missing. Because of socioeconomic and technological developments and advances in medicine, several current diagnostic modalities and treatments were previously unavailable. This limited the factors analyzed and discussed in this research as well as the interpretation of the research findings. Future research can further explore the relevant aspects that were not a part of this study. The morbidity data included in this study were limited to 12 sentinel hospitals within Wuwei City, which also limited the demographic representation of the field of study. Future research can include more provinces or be nationwide studies, thus further exploring the regional distribution and temporal trend of liver cirrhosis in greater detail.

## Conclusion

Liver cirrhosis is a chronic disease with a poor prognosis and has the potential to cause great burden to society. Our findings herein showed that unlike some other provinces and regions of China, Wuwei City’s liver cirrhosis incidence rate showed a slow but insignificant decline during the study period. Liver cirrhosis is more common in men than in women in Wuwei City, and the difference in the trend between men and women was statistically significant. Peasants are affected by liver cirrhosis the most, but in recent years, the composition ratio of workers and other occupations has been increasing. The overall standardized liver cirrhosis rate increased from 1995 to 2016 at an average rate of 16.7% per year, and although the increasing growth was evident from 2010 to 2013, after 2013, the growth rate slowed down. This decline may be attributed to China’s hepatitis B prevention and control policies. The rate of liver cirrhosis is increasing faster in women than in men, and therefore, it is necessary to strengthen the diagnosis, treatment, prevention, and control measures for diseases related to liver cirrhosis. Our findings can help make the regional prevention and treatment programs on liver cirrhosis more effective and may help factor in environmental aspects and geographical characteristics in high-incidence areas for better planning to counter this disease. The incidence of cirrhosis has obvious aggregation, and therefore, the prevention and control policies for liver cirrhosis and related diseases should not be based on administrative divisions but rather on regional prevention and control policies.

## Data Availability

The raw data supporting the conclusion of this article will be made available by the authors, without undue reservation.

## Abbreviations

APC, Annual percent change; AAPC, Average annual percent change; CI, Confidence interval; HbsAg, Hepatitis B virus surface antigen; HBV, Hepatitis B virus; HCC, Hepatocellular carcinoma; IL, Interleukin; NRCMS, New rural cooperative medical care system; PHC, Primary hepatic carcinoma; RR, Relative risk; SD, Standard deviation.
